# Commercially Available Ion-Releasing Dental Materials and Cavitated Carious Lesions: Clinical Treatment Options

**DOI:** 10.3390/ma14216272

**Published:** 2021-10-21

**Authors:** Amel Slimani, Salvatore Sauro, Patricia Gatón Hernández, Sevil Gurgan, Lezize Sebnem Turkun, Ivana Miletic, Avijit Banerjee, Hervé Tassery

**Affiliations:** 1 LBN/Faculté d’Odontologie, Université de Montpellier, 34193 Montpellier, France; amel.slimani@umontpellier.fr; 2 Dental Biomaterials and Minimally Invasive Dentistry, Department of Dentistry, Cardenal Herrera-CEU University, CEU Universities, 46115 Valencia, Spain; salvatore.sauro@uchceu.es; 3 Department of Therapeutic Dentistry, I. M. Sechenov First Moscow State Medical University, Moscow 119146, Russia; 4 Department of Odontostomatology, University of Barcelona, 08007 Barcelona, Spain; patricia@pgaton.com; 5 Department of Restorative Dentistry, Faculty of Dentistry, Hacettepe University, Ankara 06100, Turkey; sgurgan@gmail.com; 6 Ege University School of Dentistry, Izmir 35040, Turkey; sebnemturkun@gmail.com; 7 Department of Endodontics and Restorative Dentistry, School of Dental Medicine, University of Zagreb, Gunduliceva ul. 5, 10000 Zagreb, Croatia; miletic@sfzg.hr; 8 Conservative & MI Dentistry, Faculty of Dentistry, Oral & Craniofacial Sciences, King’s College London, London SE1 9RT, UK; avijit.banerjee@kcl.ac.uk; 9 Ecole de Médecine Dentaire de Marseille, Université d’Aix-Marseille, 13385 Marseille, France

**Keywords:** ion-releasing dental materials, high/low patient caries risk, cavitated carious lesions

## Abstract

The contemporary approach for operative caries management emphasizes personalized interventions for each patient, dependent upon the individual’s caries susceptibility/risk, the stage of the carious lesion and its activity. The clinician’s challenge is to optimize the extent of cavity preparation and the choice of dental restorative biomaterials, appreciating the benefits offered by ion-releasing restorative materials. There is a growing application of bioactive/bio-interactive materials in minimally invasive operative dentistry, as they may help with tissue recovery by ion release. In case of moderate or extensive occlusal cavitation, the clinical criteria include the individual caries susceptibility and carious lesion activity. In high caries risk cases, ion-releasing biomaterials (IRB) can be used, as well as for active carious lesions. In proximal lesions, the clinical criteria include the individual caries susceptibility, the lesion activity and presence of cavities with little or no enamel at the gingival margin. This article aims to discuss the restorative ion-releasing options, according to different clinical situations, and the caries susceptibility to manage cavitated carious lesions in permanent adult teeth.

## 1. Introduction

Minimum intervention, minimally invasive and preventive dentistry are defined and conceptualized in the form of concepts such as Caries Management by Risk Assessment (CAMBRA^®^), CariesCare International^®^ and the minimum intervention oral healthcare delivery framework (MIOC) [1,2,3,4,5,6,7,8,9,10].

Dentists, dental therapists and members of an oral healthcare team should all keep up to date with the contemporary caries management and the requirements and opportunities given by new developments in dental materials. This implies individualized patient care delivery with responsibilities from the oral healthcare team and patient, using “up to date” methods for detection and diagnosis of conditions, prevention and control, minimally invasive operative management and suitable personalized recall strategies, all to maintain lifelong oral health. This minimum intervention oral healthcare (MIOC) delivery framework includes the clinical domain of minimally invasive dentistry (MID), with three levels of intervention: non-invasive, micro-invasive and minimally invasive [4,11]. All should be biological, respecting oral hard and soft tissues and mastering the use of contemporary technologies and bioactive/bio-interactive “smart” materials. Nevertheless, clinical approaches vary depending on location and remains subject to the ethics of the practitioner and the health policies of each country concerned. Approximately 60% of the 170 million resin composite and dental amalgam restorations placed annually in the United States are replaced due to failed restorations [12]. Resin composite restorations fail at 2–3.5 times the rate of dental amalgam. Each subsequent re-restoration risks pulp injury, increased tooth tissue destruction and eventually, tooth loss. High-risk patients and patients with advanced carious lesions are particularly vulnerable to restoration failure [13]. The compromised effectiveness of dentine adhesives is particularly problematic for gingival margin lesions, which typically have very little enamel present for bonding. Restorations at this margin are particularly prone to secondary lesion development, due to difficulties in obtaining adequate moisture control. Indeed, 80–90% of secondary caries is located at the gingival margin of Class II and Class V restorations [14]. Kreth et al. [15] have outlined the features required of future dental biomaterials that should improve the situation in these difficult operative areas: (a) chemical modification (cell membrane disruption, antifouling); (b) topography: surface patterning (engineered chemical nano-topographies, photo-induced mechanical bacterial release); (c) chemical-releasing materials (e.g., chlorhexidine (CHX), triclosan, silver particles, doped adhesives, nitric oxide-releasing silica nanoparticles).

There remains a need to validate new strategies with existing products on the market, to define ion-releasing biomaterials (IRB), outline reasons to intervene and when to use such IRB instead of a conventional resin composite. As remaining dentine thickness overlying the pulp cannot be accurately assessed clinically, the use of a biologically based material may be recommended, such as a hydraulic calcium silicate or glass-ionomer cement, which could be applied as a protective layer prior to definitive restoration with a resin-based composite restoration [16,17]. Moreover, conventional resin composites lack the ability to increase the local pH, which can allow the growth of more acidogenic/aciduric bacteria, therefore developing a more cariogenic overlying biofilm. Together with a lack of antibacterial properties, a lack of buffering may account for the higher susceptibility of resin composites to secondary caries [18].

The aim of this article is to discuss the use of IRB in the minimally invasive operative management of cavitated carious lesions and introduce the Bioactive Dental Concept as a clinical guide (see therapeutic options sections) when using IRB.

## 2. Definition of IRB

Larry Hench has described a bioactive material as one that elicits a specific cellular and biological response at the interface of the material, which results in the formation of a bond between the tissues and the material or one that forms a surface layer of an apatite-like material in the presence of saliva or a saliva-like substitute [19,20]. The vast majority of biomaterials in dentistry do not meet this ‘bioactive’ definition; others use the term ‘bio-interactive material’, which is ideally able to bind to collagen, acting as a template of calcium and phosphorus and stimulating the nucleation of apatite crystallization, protecting collagen from degradation, providing an adequate pH to favor new mineral formation and repelling or constraining bacteria [21].

## 3. Reasons and When to Intervene

Recommendations were clearly defined in an expert Delphi consensus statement, and decision-making involves three criteria: cavitation, caries activity and cleansability of the carious lesion [22].

The use of IRB (occlusal or proximal lesions) can be considered when facing these clinical situations:

- Patient with a high individual caries risk: to favor the caries risk rebalance [3,23,24,25,26,27]. The microbiological diagnosis can be part of the patient caries risk and the individual caries assessment is mainly based on protective factors (e.g., regular preventive oral care), patient factors (e.g., visible old dental plaque) and clinical finding (e.g., approximal carious lesion).
- Active carious dentine lesion: to promote caries reversion, buffering effect and interdiffusion zone with a remineralization potential [28].
- A cavity with little or no enamel at the gingival/peripheral margins, in addition to the carious lesion activity [29,30].

## 4. Therapeutic Options for Excavation and Tissue Conditioning

**Figure 1:**
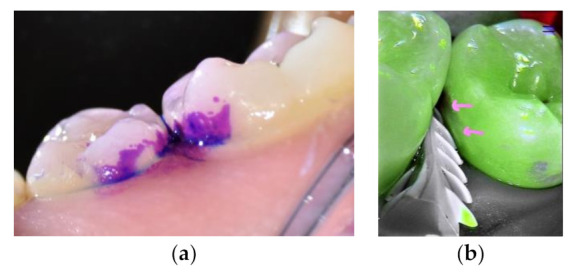
Premolars before and after cleaning using EMS biofilm discloser and an airflow with erythritol powder. (**a**) Biofilm daylight image after the application of a plaque disclosing dye (Soprolife^TM^ camera). (**b**) An enamel white spot revealed after plaque cleaning (pink arrows, fluorescence image, Soprolife^TM^ camera).

- To improve carious lesion assessment and its treatment, cleaning the tooth is recommended. One simple and easy way to clean the tooth is the use of an airflow device (e.g., erythritol powder or soft sodium bicarbonate) to remove the biofilm and debris without damaging the remaining hard tissues (Figure 1) [31]. Prophylactic paste applied with rotative brushes could interfere with the photonic signal of visual aids [31,32].

- Magnification and photonic signals such as fluorescence, infrared or photothermal radiometry may help to re-evaluate the caries diagnosis and caries activity [31,33].
- Peripheral seal concept: This procedure improves the sealing of the border and may preserve the inner dental tissue. Ceramic or polymer burs could help to selectively remove the carious tissue to hard dentine (on border, 2 mm width) and preserve the gingival enamel margin. In particular, polymer burs are offering a compromise between the caries removal effectiveness (CRE) and minimal invasiveness potential (MIP) to remove the soft carious tissue [34,35,36].
  ○ If moderate (ICCMS score 3–4) carious lesion: excavation to firm or leathery dentine is recommended according to the clinical depth of the carious lesion [22].
  ○ If extensive (ICCMS score 5–6) carious lesion: excavation to soft dentine could help to reduce the risk of vital pulp injury [22].
- Options:
  ○ Sonic and ultrasonic abrasion can be used only to shape the cavity [37].
  ○ Sodium bicarbonate, glycine or erythritol are efficient for biofilm removal, but not for the excavation steps.
  ○ Hand excavation with spoon-shaped excavators could help during the selective caries excavation [38].

Different options can be considered for cavity conditioning before adhesive application:

- Air-abrasion using Bioglass 45S5 to induce a therapeutic “bio-interactive’’ smear layer to protect the bonded interface and preserve adhesion performance [21,39].
- Chlorhexidine (CHX 2%, 1 min, no rinsing) has been recommended for its antibacterial effect and its action against MMPs [40]. However, its use is still controversial in regard to long term result [2,41], but leathery or firm carious dentine remain infected and the remnant bacteria in the dentine could provoke subclinical pulpal inflammation over time. That raises questions about carious dentine disinfection [42].
- Photo-active disinfection (PAD) as an antimicrobial aid using photoactive compounds (e.g., 1 min of tolonium chloride, then 1 min of light activation with a specific wavelength and rinse) to produce oxygen-based free radical under a light source. Its efficiency is reduced as the “reactive oxygen species” diffuse more or less around 100 nm with a very short half-life. However, PAD remains a promising technique in restorative and periodontal treatments [43,44,45].
- Sodium hypochlorite (NaOCl): Replacing traditional acid dentine conditioning with a 10% polyacrylic acid solution, before GIC application, a deproteinization and antibacterial step with NaOCL 6% for 15 sec can be used. The general appearance of the hybrid layer was maintained after deproteinization, even with 10% NaOCl gel subject to not exceeding 30 s of application [46,47,48].
- Chemo-mechanical caries removal agents (CMCR) or enzyme treatment, such as a gel (Papacaries), are applied before self-etch or after etching and rinse for its antibacterial effect and may help to selectively excavate infected dentine in combination with specific smooth hand excavators [49,50].
- Dentine surface treatment with 37% phosphoric acid for 5 s has no negative effect on bonding of RM-GICs adhesion to dentine compared with using polyacrylic acid for 10 s [51,52].
- Silver Diamine Fluoride (SDF) under an HV-GICs restoration. The combination of SDF and CMRC or Papain enzyme would enhance the anti-bacterial effect [53].
- Adhesive system options including antiseptic molecules:
- Adhesive systems with 0.2% of CHX having anti-MMPs activities or including remineralizing modified calcium phosphate [40,54,55].
- Adhesive systems with antibacterial monomer 12-methacryloyloxydodecylpyridinium bromide (MDPB): antibacterial activity based upon MDPB against S. mutans, L. casei and A. naeslundii, and the ability to disinfect cavities containing residual bacteria [54,56,57].

If inactive caries:

No specific recommendations in term of tissues conditioning. Hard, black residual dentine must be monitored.

As previously introduced, the Bioactive Dental Concept aims to guide the general practitioner clinically, with simplified flow charts based on three different clinical recommendations.

- Individual caries risk assessment evaluation is mandatory.
- Magnifying and using photonic signals to evaluate the caries score and caries activities could be useful.
- Use bioactive materials according to the caries activity, the individual caries risk assessment, the available enamel amount in gingival margins and accessibility of the lesion (Table 1).

**Table 1:** Bio-interactive materials with their properties and commercially available brands (at the time of publication).

Types of Materials	Bio-Interactive Properties	Biological Effects	Drawbacks	Commercially Available Products
**Conventional GICs/Glass polyalkenoates**	Ions released: F, Ca and Al Formation of polyalkenoate salts with a interdiffusion zone and calcium polycarbonate. * Flowable GIC with higher fluoride release	Antibacterial effects, hard tissues remineralization, bulk-fill reaction	Long setting reaction, low wear resistance, esthetic	IonoStar Plus, IonoFil, Aqua Ionofil Plus Plus (VOCO, Cuxhaven, Germany), Ketac Universal, Ketac Fil Plus (3M ESPE, St Paul, MN, USA). Riva Self Cure, Riva protect * (SDI, Victoria, Australia). * Fuji Triage (GC, Tokyo, Japan)
**High-viscosity GICs**	Ions released and reloaded: F, Ca, Al. Formation of polyalkenoate salts with an interdiffusion zone and calcium polycarbonate	Antibacterial effects, hard tissues remineralization, bulk-fill reaction	Short setting reaction, high viscosity depends on products	Fuji IX (Fast, Extra), (GC, Tokyo, Japan). Chemfil Rock (Dentsply, York, PA, USA), IonoStar Molar, Ionofil Molar, Ionofil Molar AC Quick (VOCO, Cuxhaven, Germany), Ketac Molar, Ketac Molar Quick, (3M ESPE, St Paul, MN, USA). Riva self-cure HV (SDI, Victoria, Australia)
**Glass hybrid cements**	Ions released and reloaded: F, Ca, Al. Formation of polyalkenoate salts with an interdiffusion zone and calcium polycarbonate	Antibacterial effects, hard tissues remineralization, bulk-fill reaction	Short setting reaction, high viscosity depends on products	Equia Forte, Equia HT (GC, Tokyo, Japan)
**RM-GICs and RM-GICs HV**	Ion released: F, Ca, Al. Formation of polyalkenoate salts with interdiffusion zone. Formation of calcium polycarbonate	Facilitates tissues remineralisation, antibacterial effects	Not a true bulk-fill reaction, no covalent or ionic bond between the 2 networks, absorption of water due to residual HEMA, low wear resistance, except RMGICs-HV	Fuji II, Fuji II LC (GC, Tokyo, Japan), Ionolux (VOCO, Cuxhaven Germany), Photac Fil Quick Aplicap, Ketac Nano, Vitremer (3M ESPE, St Paul, MN, USA), Riva Light-cure, Riva Light Cure HV, (SDI, Victoria, Australia)
**Mineral-enriched resin composite**	Release of F, powder containing fluoro-alumino silicate particles and polyacid components	Material * can reduce the degradation during load cycling and/or prolonged storage in artificial saliva of the hybrid layer created with modern universal adhesive applied in etch and rinse mode	Lack of studies for Re-Gen products	Activa, Activa liner *, Presto * (Pulpdent, Watertown, NY, USA), Re-Gen Flowable Composite, Re-Gen Bulk Fill Composite (Apex, Las Vegas, NV, USA), Replica bulkfil (Parkwell, Boston, MA, USA)
**Mineral-enriched self-adhesive resin composite**	High molecular weight polyacrylic acid functionalized with polymerizable groups (MOPOS). Photo and chemical activation. F and Al ions release. No adhesive system combined	Release of F, C and Al.	Very high viscosity, lack of evidence as new product, short time setting. Lack of studies	Surfil 1 Self-adhesive hybrid resin composite (Dentsply, York, PA, USA)
**Giomers**	Resin composite materials where a pre-reacted glass-ionomer (PRG) filler technology has been incorporated	The main advantage of this material would be its improved F release, but otherwise their clinical performance can be compared to conventional resin composites	To be used as resin composite for restorative dentistry	Beautifil II, Beautifil II Gingiva Shades, BeautiSealant (Shofu Dental Corporation, Kyoto, Japan)
**Mineral-enriched Alkasite resin composite**	No acid/base reaction. Alkaline glass filler reacting with water. In this SiO2, 3 salts are connected (Na_2_O, CaO, CaF_2_). In contact with the saliva these salts are dissolved and released Ca, F and OH ions depend on the pH. Combine with a specific primer.	Hydroxyl ion: regulates the pH-value during acid attack and prevent demineralization. Buffering ability at pH 5.7F and Ca: to prevent demineralization of the tooth substrate. Forming apatite in vitro on dentine at pH 7 if phosphate available	Very high viscosity, lack of evidence as new product, short time setting. Lack of studies	Cention N (Ivoclar-Vivadent, Schaan, Liechstenstein)
**Calcium silicate-based**	These materials set by a hydration and precipitation mechanism. The remineralisation mechanism is based on an alkaline reaction. The alkaline setting reaction of these cements can reduce MMP activity and also has beneficial antibacterial effects on caries-affected (and infected) dentine	Degradation of collagen fibrils occurs and leads to the formation of a porous structure, which facilitates the penetration of high concentrations of Ca and carbonate ions, leading to increased mineralisation in the interface zone	Time setting very long. Liner or temporary restoration	Biodentine (Septodont, St Maures des fossés, France)
**OKResin-modified MTA**	Bio-interactivity principles close to the calcium silicate based material but less effective	Vital pulp therapy. Easy to use, dentine bridge formation	Used as liner if close to the dental-pulp complex	TheraCal LC (Bisco, Schaumburg, IL USA), MTA Plus, Neo MTA (Avalon Biomed Inc., Houston, TX, USA), Endosequence BC sealer (Brasseler, Savannah, GA, USA), Angelus MTA, MTA Bio (Angelus, Londrina, Brazil). BioAggregate^®^ (Innovative BioCeramix), RetroMTA and BioMTA, (IBC, Vancouver, BC, Canada)
**Silver Diamine Fluoride**	Silver is an anti-microbial agent. F has bacteriostatic effect and potassium iodide used in conjunction with SDF provides a powerful antimicrobial effect as well as reducing potential staining of teeth	High caries risk, geriatric dentistry. Apply before HV-GIC. Can be combined with enzyme or chemo mechanical conditioning	Discoloration. A new version with water solution (e.g., Riva Star Aqua) could reduce this drawback.	Riva Star, Riva Star Aqua (SDI, Victoria, Australia), Cariestop (Biodinâmica, Ibipora, Brazil), FAgamin (Tedequim, Cordoba, Argentina), Advantage Arrest (Elevate Oral Care, West Palm Beach, FL, USA), e-SDF (Kids-E-Dental, Mumbai, India), Saforide (Toyo Seiyaku Kasei Co. Ltd., Osaka, Japan)
**Resin-modified glass-ionomer** **adhesives**	Ionglass™ fillers, which contain fluoro-aluminosilicate glass for radiopacity and F release.	With composite restoration alone or combined with RM-GICs as dentine		Riva Bond LC™ (SDI, Victoria, Australia)
**Adhesive with CHX**	Release of CHX	Antibacterial effects, stabilization of the hybrid layer, anti-MMPs effects	Time limiting effects	Peak adhesive (Ultradent, South Jordan, UT, USA)

* was related to “Flowable GIC with higher fluoride release” in the column “Bio-intercative properties”.

Therapeutic Options for Cavitated Occlusal Lesions.

For occlusal lesions, clinical difficulties are reduced to three points as the enamel border surrounds the entire cavity preparation:

- Individual caries susceptibility/risk (ICR): high caries risk (HCR), low caries risk (LCR).
- Carious lesion activity.
- Carious lesion extension: moderate or extensive.

Moderate carious lesion:

Excavation: extended to the firm or leathery dentine [2,58].

High Caries Risk (HCR): recommended IRB:

○ Conventional GICs.
○ Flowable conventional GICs: high fluoride ion releasing for a provisional restoration.
○ High viscosity GICs self or light cure [59].
○ Mineral-enriched alkasite resin composite, even the lack of studies, this material is considered as bioactive [60].

LCR + active carious lesions: IRB as dentine substitute.

○ Option 1: if RM-GICs: apply “dentine conditioner”, rinse then inject RM-GICs as dentine substitute.
○ Option 2: if RM-GICs or RM-GICs-based resin composite (mineral-enriched resin composite): can be used with universal adhesive in etch-rinse* (5 s selective etching) may contribute to maintain the bonding performance [61].
○ Option 3: if HV-GICs (self or light cure): apply with “dentine conditioner”, (see optional recommendations), rinse then inject HV-GICs.
○ Option 4: if the bottom of the preparation remains far from the dental-pulp complex, traditional restoration with resin composite can be used.

LCR + non-active caries: all restorative techniques are available (flow, low flow, dual, conventional/warmed resin composite).

Extensive and active occlusal carious lesions

HCR: high viscosity GICs self or light cure (combined or not with SDF) or calcium silicate-based material.

**Figure 2:**
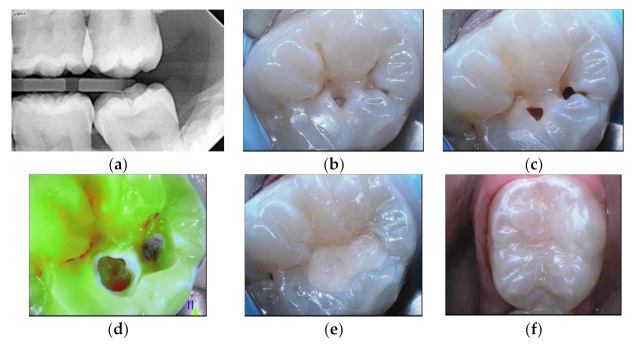
Lower molar with extensive occlusal cavities. (**a**) Preoperative radiograph. (**b**) Daylight image before cleaning. (**c**) Tooth after cleaning with sodium bicarbonate airflow. (**d**) Fluorescence image of the tooth during cavity preparation (Soprolife^TM^ camera). (**e**) GIC application as a liner. (**f**) Resin composite restoration.

LCR + active lesion (Figure 2): IRB as dentine substitute.

○ In case of pulp proximity, consider applying a bioactive liner like calcium silicate-based material [16,62].

LCR + inactive lesion: all restorative therapeutic options are available (flow, low flow, dual, conventional/warmed resin composite).

Therapeutic Options for Cavitated Proximal Lesions

Clinical Criteria:

- Caries risk.
- Carious lesion activity.
- The presence or not of bondable enamel at the gingival margins.

**Figure 3:**
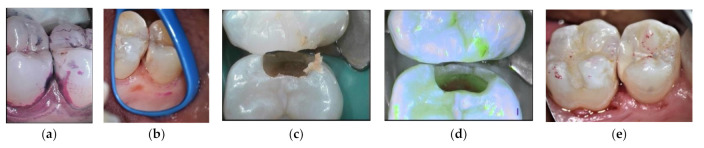
HCR patient treated with HV-GIC LC: (**a**) biofilm disclosing step; (**b**) visible cavitation after air flow cleaning; (**c**) cavity preparation; (**d**) fluorescence image after cavity preparation: red signal of the leathery dentine (Soprolife^TM^ image); (**e**) HV-GIC LC restoration.

**Figure 4:**
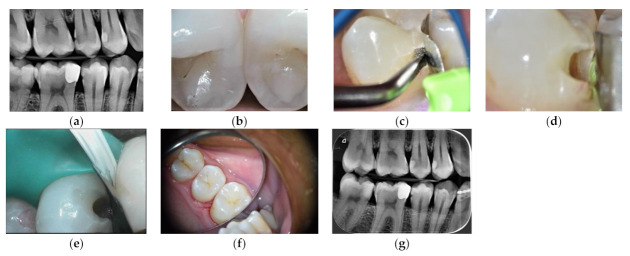
Slot cavity preparation on a distal carious lesion of an upper premolar restored with HV-GIC LC: (**a**) pre-operative radiograph; (**b**) marginal crest modification white spot (blue arrow); (**c**) US diamond insert half-round shape; (**d**) slot preparation; (**e**) metal matrix setting; (**f**) final occlusal view; (**g**) post-operative radiograph.

Moderate lesion with bondable enamel:

- HCR: see therapeutic options for occlusal lesions for HCR patients (Figure 3).
- LCR:
  - Option 1: If a slot or a tunnel preparation (Figure 4), the use of IRB for active and non-active carious lesions remains mandatory. Prefer RM-GICs or HV-GICs light cure, as it is easier to use due to the longest setting time and easy removal in case of overflow. The occlusal increment of the tunnel restoration is preferably covered with a resin composite after adhesive procedures.
  - Option 2: if conventional preparations and inactive carious lesion, all technics are possible, and IRB is preferred as dentine substitute in case of active caries depend on the residual dentine thickness.

**Figure 5:**
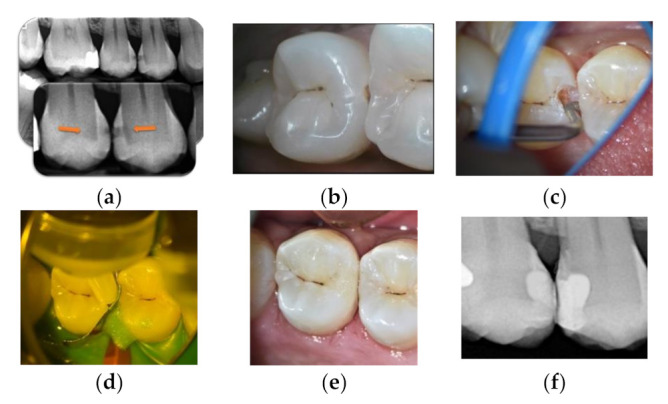
Mineral alkasite-enriched composite obturation: (**a**) pre-operative radiograph; (**b**) cavitation visible after cleaning steps; (**c**) sonic curved insert; (**d**) Cention^®^ injection; (**e**) final view in daylight; (**f**) post-operative radiograph.

Extensive lesion with enamel present in gingival margin (Figure 5).

See therapeutic options cited for an extensive occlusal lesion.

Clinical criteria:

- Caries risk.
- Caries lesion activity.
- Enamel available in gingival margins.

Extensive lesion with no bondable enamel in gingival margin

- HCR: favor HV-GICs (self or light cure) to support the cavity constraints or apply a calcium silicate-based material in case of pulp proximity [59,60].
- LCR: IRB as dentine substitute for both active or inactive caries [12].
  - Option 1: RM-GICs or HV-GICs (self or light cure), mineral enriched resin composite.
  - Option 2: mineral-enriched-alkasite resin composite or mineral enriched self-adhesive resin composite only (new products with lack of evidence).
  - Option 3: Calcium silicate-based or resin modified MTA.

## 5. Discussion

### Clinical and Scientific Considerations

Consensus recommendations on tissue removal and lesion cavity management have been published by the International Caries Consensus Collaboration (ICCC^TM^) [22,58]. The sealing quality of a restoration is subject to the quality of the dental tissue surface. Gaining an adequate peripheral seal inactivates the retained bacteria and preserves affected, non-demineralized and remineralizable tissues to obtain an adhesion-friendly substrate. This aims to achieve successful restoration placement and protection of the dentine–pulp complex to favor the combination of hand excavation with chemo-mechanical gels/solutions [37].

Adjunct technologies can help discriminate the nature of the carious tissue. Intrinsic fluorescence signals from carious dentine can guide tissue removal; necrotic, caries-infected dentine appears dark green, while the leathery, caries-affected dentine is grey green with red shadows. This approach is conducted to optimize the restoration peripherical sealing and therefore avoid bacterial infiltration, in addition to promoting dentine healing using an IRB [31,32,35]. For teeth with shallow or moderately deep lesions, selective removal to firm dentine excavation protocols should be followed. In contrast, in deep lesions (radiographically extending into the inner third of dentine) in permanent teeth, selective removal to soft dentine should be performed, assuming pulp sensibility tests are positive. For extensive deep carious lesions, an IRB like GICs or calcium silicate-based materials as pulp protection can be used to encourage dentine remineralization and pulp sensibility maintenance [16,17].

BAG (Bioglass 45S5-BAG or fluoride-containing phosphate-rich bioactive glass (BAG-F)) could be used prior to a restoration as a tissue surface conditioner or included directly in the composition of the restorative/adhesive system. The use of BAG as a tissue conditioner produces a healthy enamel surface minimally invasively, and it is an interesting strategy to create a modified smear layer within the interface, as it seems to induce remineralization and protection of the dentine bonded interface [39]. BAG could be applied externally via an air-abrasion device or included directly in the composition of the restorative/adhesive [63,64,65,66,67].

The evidence supporting the clinical efficacy of cavity disinfection is limited in the routine operative caries management protocols [68]. In daily practice, this could be achieved after dentine conditioning or before in the case of self-etch systems. Moreover, some biomaterials incorporate bioactive molecules, aiming to improve tissue disinfection. Some in vitro studies have shown the benefits of caries disinfection by bacterial reduction and MMP inhibition [69,70,71]. Silver diamine fluoride (SDF) has antibacterial properties, and current investigations are aimed towards the reduction of the oxidative tissue staining found after SDF application. A saturated potassium iodide solution has been developed to reduce staining after the use of SDF and before the restoration placement using GIC. Particular attention must be given to the use of SDF before a universal self-etch mode adhesive system, as it could reduce the dentine bonding stability [53]. Cavity shaping is more appropriate using sonic and/or ultrasonic abrasion, but one should be aware that they tend to underprepare the cavities in the presence of soft carious tissues. Air-abrasion is assimilated to hand excavation for the time required and amount of dentine removed, but its efficiency depends on the powder’s hardness. Conventional hand excavation appeared to offer the best combination of efficiency and effectiveness for carious dentine excavation [32,37,70].

There is no ideal bioactive restorative material with biological properties for tissue recovery and optimal optical and mechanical properties. However, ionic dissolution from ion-releasing materials may be the key factor in unlocking their remineralization potential. Calcium and phosphorous are the main components of the biological apatite. Other inorganic ions, such as fluoride, zinc, magnesium and silanol groups, may also act as substitutes in apatite crystal formation. These materials may offer adjunctive strategies for the treatment of cavitated carious lesions: (1) deliver mineral ions, in order to induce in situ remineralization, pulp protection, stabilization of the hybrid layer and act as a template of calcium and phosphorus, stimulating nucleation of apatite crystallization; (2) protect collagen from degradation: pH buffering effect, protection from MMPs attack and preservation of the hybrid layer and therefore a reduction of microleakage; (3) induce a pH to favor a buffering effect and new mineral deposition; (4) repel or constrain bacteria [21]. Understanding the biological concepts of bioactive material categories may help the clinician to use the proper material, but also to recognize the advantages and the limitations of each material according to each specific clinical case. The effects of ion-releasing biomaterials on general health need more investigation, with a particular attention on oral soft tissues, stem cells and biofilm formation [71,72,73,74].

## Data Availability

Not applicable.
